# Air pollution modifies colonisation factors in beneficial symbiont *Snodgrassella* and disrupts the bumblebee gut microbiome

**DOI:** 10.1038/s41522-024-00632-3

**Published:** 2025-01-02

**Authors:** Hannah R. Sampson, Natalie Allcock, Eamonn B. Mallon, Julian M. Ketley, Julie A. Morrissey

**Affiliations:** 1 Department of Genetics and Genome Biology, University of Leicester, Leicester, UK; 2 Electron Microscopy Facility, Core Biotechnology Services, University of Leicester, Leicester, UK

**Keywords:** Microbiome, Biofilms

## Abstract

Particulate air pollutants, a major air pollution component, are detrimental to human health and a significant risk to wildlife and ecosystems globally. Here we report the effects of particulate pollutant black carbon on the beneficial gut microbiome of important global insect pollinator, the buff-tailed bumblebee (*Bombus terrestris*). Our data shows that exposure to black carbon particulates alters biofilm structure, gene expression and initial adhesion of beneficial bee gut coloniser, *Snodgrassella alvi*. Exposure of adult *Bombus terrestris* to non-toxic black carbon particulates significantly increased viable bacteria on MRS agar and 16S absolute abundance of beneficial bacteria *Bombilactobacillus* in Post-treated bumblebees compared to Pre-treated, demonstrating disruption of the bumblebee gut microbiome. These findings show that black carbon exposure has direct, measurable effects on bees’ beneficial commensal bacteria and microbiome. Together these data highlight that black carbon, a single type of particulate pollution, is an underexplored risk to insect pollinator health.

## Introduction

Air pollution is the world’s largest environmental health risk, with over 90% of the global population residing in areas that exceed the World Health Organisation’s air quality guidelines^1^. Air pollutants originating from both natural and anthropogenic sources contaminate our atmosphere and can be transported large distances, depositing on plants, soil and water sources. Air pollution is comprised of gaseous components and solid particulate matter^2^. Particulate matter exposure is associated with increased human respiratory and cardiovascular disease severity^3^, and in the environment exposure alters ecosystem diversity, nutrient availability and is detrimental to wildlife health^4,5^.

Black carbon is a major component of particulate matter, which is produced from the incomplete combustion of fossil fuels and biomass^6–8^. Black carbon can bind with other particles acting as a carrier for other more toxic pollutants^6,7,9^. The majority of black carbon sources are anthropogenic, including emissions from industry and vehicles, natural sources include biomass burning and forest fires^9,10^. Black carbon concentrations have remained stable since monitoring began with higher concentrations typically recorded from urban roadsides^7,8,11^.

Ambient particulate pollutants are complex, typically consisting of carbon core particulates with different mixtures of metals and organics e.g., polycyclic aromatic hydrocarbons and other contaminants^12^. Exposure to particulate pollutants can damage host tissue and disrupt the immune response resulting in detrimental effects on the health of the host^13,14^. These detrimental effects on the host can change host-microbial interactions, increasing infectious disease and altering host microbiomes^13,15–18^. Notably particulate pollutants can act directly on bacteria changing their behaviour to increase biofilm formation on abiotic surfaces and colonisation of human epithelial cells and in vivo murine infection models^19–22^.

Both honeybees and bumblebees are vital to the maintenance of natural ecosystems and are essential pollinators in agriculture^23–25^ making the study and maintenance of their health a crucial area of research. Bees house a distinct, specialised community of core gut bacterial symbionts (the bee gut microbiome) that are unique to the bee gut and colony environment^26–32^. The maintenance and balance of this core gut microbial community is a key element to bee health assisting in the digestion of essential nutrients, promoting host weight gain and providing protection from pathogens and environmental stressors^26,33–35^.

Core bacterial phylotypes (*Snodgrassella, Gilliamella, Bifidobacteriaceae, Lactobacillus* Firm-4 and *Lactobacillus* Firm-5) have been identified in almost every corbiculate eusocial bee (e.g., honeybees and bumblebees) and usually make up the majority of their gut microbiome^27,28,30,32,36–38^. *Lactobacillus* Firm-4 (*Bombilactobacillus bombi*) and *Lactobacillus* Firm-5 reside in the hindgut (ileum and rectum) and play a key role metabolising pollen, providing nutrients for the host^33,34,39^. *Snodgrassella* and *Gilliamella* engage in syntrophic cross-feeding of nutrients and predominantly reside in the ileum in a biofilm structure, with *Snodgrassella* bound to the ileum epithelium and *Gilliamella* on top^33,36,40,41^. *Snodgrassella* is a key gut coloniser, able to persist in an otherwise sterile gut without other members of the gut microbiome^40^. Several genes are essential to this colonisation process including adhesins and type IV pilus genes^40,42,43^. Core bacteria interact with the bee host influencing their immune response and hormone signalling, demonstrating that core symbionts not only breakdown food but also shape the internal physiology of the host^33,34,44^.

Bumblebees hatch with few to no bacteria in their intestine and obtain their characteristic mature adult gut microbiome over four to six days through interactions with nestmates and hive material in their colony^26,45,46^. Successful establishment of the microbiota plays a major role in protecting bumblebees against pathogens^26^. Bumblebees taken away from the colony early, before their mature gut microbiome has been established, do not develop a normal gut microbiome composition and have increased infection levels^26,45^.

Once mature, the microbiome composition of indoor-reared bumblebees remains stable as they age^47^, but this mature microbiome can be perturbed by environmental factors, for example exposure to environmental metal pollutants^48^. There is a wealth of literature concerning pollinator health and pesticides, but substantially less research on air pollution and insects^49^, highlighting the need for a better understanding of how pollutants affect insect microbiomes and health.

Honeybees have long been used as indicator organisms to assess the impact and prevalence of atmospheric pollutant contamination^50^. Bees accumulate atmospheric particulates on their head, antennae, legs and wings^51,52^ and particulates also contaminate collected pollen^53^. This means that pollutants are not only in contact with bees during foraging but are present in their food stores. *Apis dorsata* (Giant Honeybee) collected from highly polluted sites were associated with significant changes to flower visitation, heart rate and survival^52^, indicating the potential systemic impact pollution has on bees, their behaviour and longevity. Understanding more about how pollution affects bees, their gut symbionts and the mechanisms involved is important to determine the full impact on bee health.

Black carbon particulates that are 99% carbon with no confounding toxic constituents e.g., metals and polycyclic aromatic hydrocarbons will be used as a model particulate air pollutant. Previous studies have shown that exposure to black carbon alters biofilm formation and human epithelial and murine in vivo colonisation of *Streptococcus pneumoniae* and *Staphylococcus aureus*^19,22^. Notably pre-exposure to black carbon, prior to inoculation significantly alters virulence gene expression and colonisation, demonstrating that *S. aureus* genetically adapts to particulate exposure^22^. This discovery demonstrates that particulate pollutants could have more, currently unknown, influences on microbial life.

Here we report that black carbon affects the behaviour of bee gut commensals and perturbs the core bumblebee gut microbiome. Model bee gut bacteria *Snodgrassella alvi* is a prominent member of the core bee gut microbiome and able to colonise an otherwise sterile gut, forming a biofilm on the surface of the ileum epithelium. We found that black carbon altered *S. alvi* adhesion and in vitro biofilm formation on abiotic surfaces. Importantly, exposure of black carbon to the native UK pollinator the buff-tailed bumblebee (*Bombus terrestris*), reared in controlled laboratory conditions, caused direct, measurable effects on the mature bee gut microbiome composition. The findings from these two parallel investigations have noteworthy implications for the impact of air pollution on bee health (Supplementary Fig. 1).

## Results

### Black carbon increases initial adhesion of the beneficial bee gut symbiont *Snodgrassella alvi*, changing biofilm formation

Previous studies have shown that exposure to black carbon changes the host colonisation of human bacterial pathogens *S. aureus* and *S. pneumoniae*^19,22^. However, the direct effect of black carbon on beneficial bacterial commensals, such as bee gut commensals, has not been established. *S. alvi* is a prominent, beneficial bee gut commensal able to survive in an otherwise sterile gut^30,40,42,44^ and is used in this study as a model bee commensal to establish whether black carbon affects core bee gut bacteria.

Black carbon concentrations used in this study reflect environmentally relevant total amounts of air pollution that biological organisms are reported to be exposed to^54^. Dose response analysis showed no significant effect (*F*_(3, 8)_ = 1.58, *p* > 0.05) on *S. alvi* growth with higher concentrations of black carbon (Supplementary Fig. 2). For in vitro experiments, 100 µg/mL of black carbon was used because it had minimal effect on *S. alvi* growth (Supplementary Figs. 2 and 3) and is consistent with previous studies^19,22^.

**Fig. 1:**
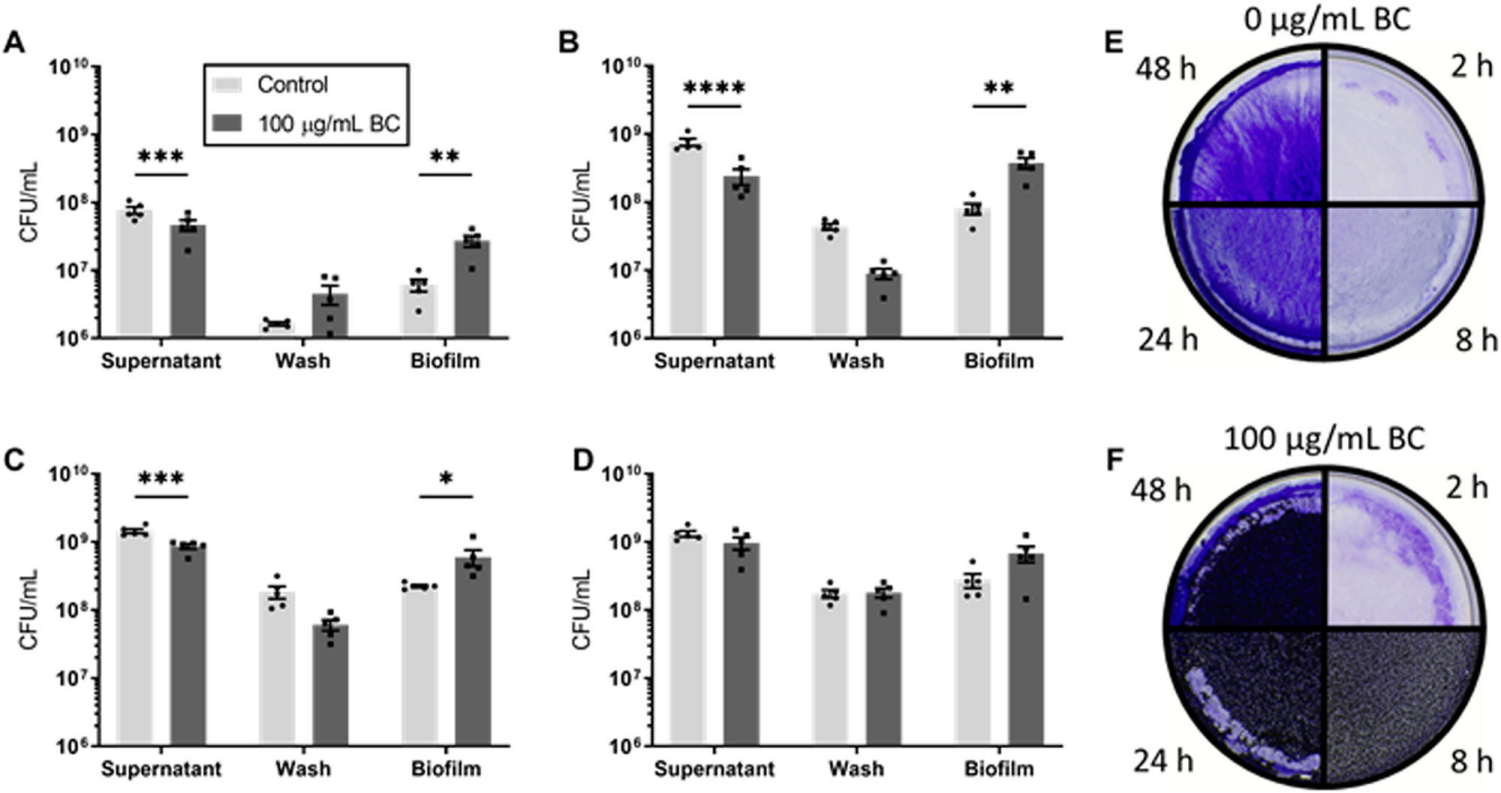
Black carbon increases *Snodgrassella alvi* early biofilm formation. *S. alvi* wkB2 was grown in BHI with 0 or 100 μg/mL black carbon (BC), aliquoted into 12 well biofilm plates and incubated at 37 °C in 5% CO_2_ conditions for 2 h (**A**), 8 h (**B**), 24 h (**C**) or 48 h (**D**). Biofilms were separated into fractions (supernatant (planktonic growth), wash (loosely adherent) and biofilm), serial diluted and plated to determine bacterial growth. Data are presented as CFU/mL and error bars represent standard error of the mean of *n* = 5 biological repeats. Two-way ANOVAs were performed followed by Šidák correction, significant differences (*p < 0.05, **p < 0.01, ***p < 0.001 and ****p < 0.0001) are indicated. Images of crystal violet stained biofilms at different timepoints treated with 0 (**E**) or 100 μg/mL black carbon (**F**).

In vivo*, S. alvi* predominantly colonises the ileum, binding to ileal epithelial cells in a biofilm structure^36,41^. The effect of black carbon on *S. alvi* wkB2 biofilm formation was measured in vitro by quantifying the viability of different fractional components of the biofilm. *S. alvi* viability was measured in three biofilm fractions; supernatant (planktonic), wash (loosely adherent) and adherent biofilm^19^. *S. alvi* biofilms exposed to black carbon had significantly higher colony forming units (CFU) at 2 h (initial adhesion) (*F*_(2, 12)_ = 20.98, *p* < 0.01), 8 h (early biofilm) (*F*_(2, 12)_ = 33.04, *p* *<* 0.01) and 24 h (early biofilm) (*F*_(2, 12)_ = 17.01, *p* < 0.05) compared to the control (Fig. 1A–C). Conversely, there were significant decreases in the CFU of black carbon treated supernatant fractions at 2 h (*p* < 0.001), 8 h (*p* < 0.001) and 24 h (*p* < 0.001) compared to the control. At the 48 h (established biofilm) timepoint, no black carbon treated fractions were significantly different to any control fractions (Fig. 1D) (*F*_(2, 12)_ = 3.484, *p* > 0.05). When the CFU of each fraction was combined, there was no significant difference between control and black carbon treated *S. alvi* total CFU (Supplementary Fig. 4) (*F*_(7, 39)_ = 33.97, 2 h *p* > 0.05, 8 h *p* > 0.05, 24 h *p* > 0.05, 48 h *p* > 0.05), therefore black carbon changed *S. alvi* biofilm behaviour but not overall growth in these conditions.

Biofilms treated with 0 µg/mL or 100 µg/mL of black carbon were visualised with crystal violet staining (Fig. 1E, F). Biofilm quantification with crystal violet was impeded by black carbon but demonstrates the clear incorporation of black carbon into the biofilm from 8 h. No black carbon was observed in the biofilm at 2 h even though there is a significant increase in viable cells in the biofilm fraction (Fig. 1A) suggesting that the increase in early adhesion is dependent on black carbon altering bacterial behaviour. Together these data show that black carbon significantly increases *S. alvi* wkB2 adherence in the early phases of biofilm formation.

### The effect of black carbon particles on *Snodgrassella alvi* biofilm structure

**Fig. 2:**
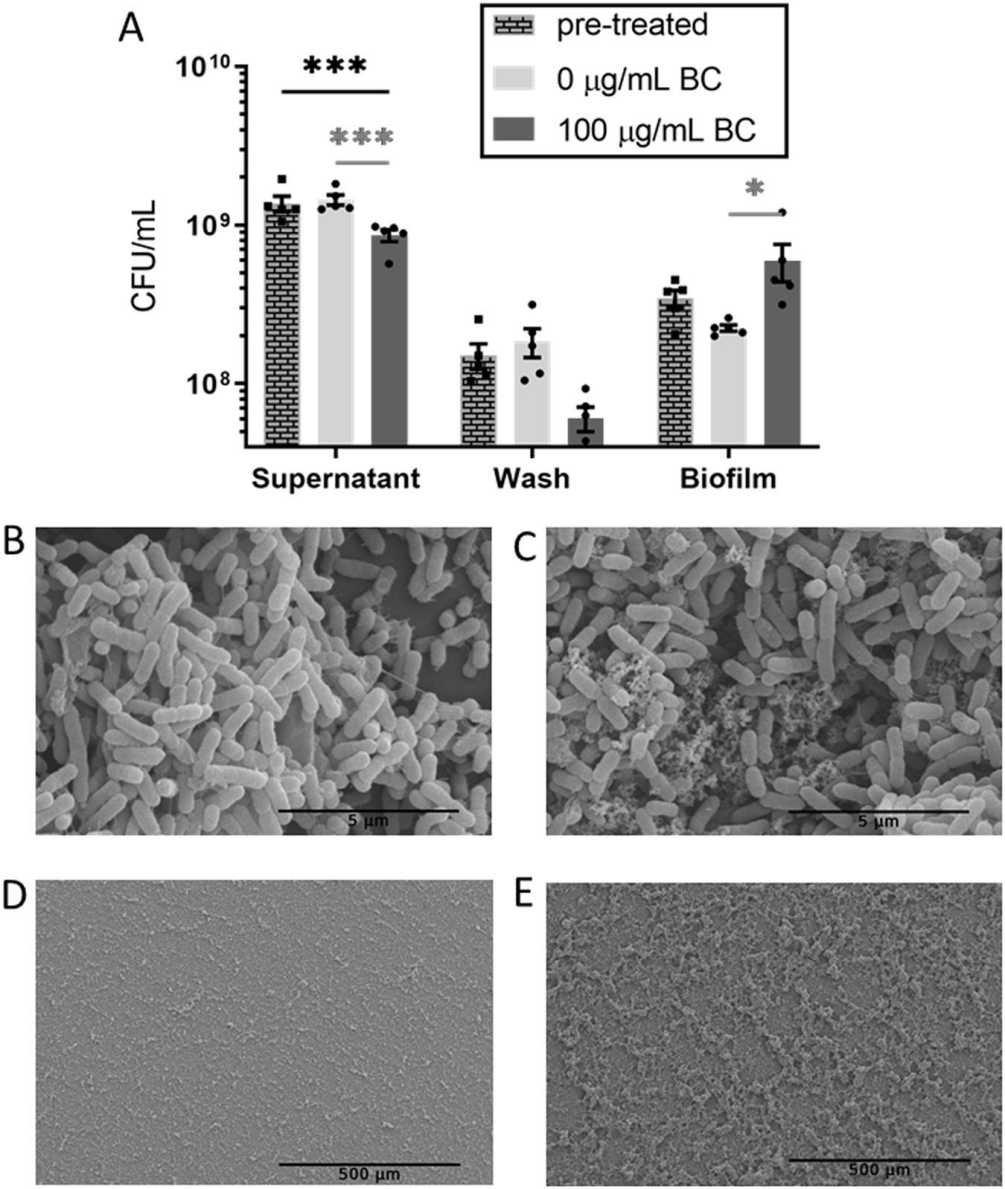
Black carbon particles change *Snodgrassella alvi* biofilm structure. **A** Pre-treated CFUs are presented alongside 0 or 100 μg/mL black carbon (BC) 24-h biofilms, biological repeats *n* = 5. Error bars represent standard error of the mean. Two-way ANOVA with Šidák correction was performed and significant differences (*p < 0.05 and ***p < 0.001) are indicated in black between the pre-treated condition and 0 or 100 μg/mL black carbon, and in grey between 0 or 100 μg/mL black carbon conditions. Scanning electron microscopy images of 24-h *S. alvi* wkB2 biofilms grown in BHI with 0 μg/mL black carbon (**B**, **D**) or 100 μg/mL black carbon (**C**, **E**).

To determine if the physical nature of the black carbon signal results in a biofilm change, *S. alvi* biofilms were grown for 24 h in BHI media that was pre-treated by adding black carbon to the media, then removing the particles, <0.2 μM by filtration, prior to *S. alvi* inoculation. The effect of pre-treated media on *S. alvi* 24-h biofilm formation was compared to black carbon treated and control biofilms. *S. alvi* biofilm formation in black carbon pre-treated media showed a similar response to the control at 24 h, with a significantly higher supernatant CFU (*F*_(4, 36)_ = 8.28, *p* < 0.001) in both control and pre-treated media conditions compared to the black carbon treated condition, but a non-significant change in biofilm CFU (*p* > 0.05) (Fig. 2A). Only *S. alvi* biofilms grown in the presence of black carbon particles had a significantly altered biofilm structure (*p* < 0.05) therefore, the physical presence of black carbon particles is required for these *S. alvi* biofilm alterations.

To establish what architectural and structural biofilm changes occur in the presence of black carbon, scanning electron microscopy was conducted on *S. alvi* biofilms. Biofilms were grown for 24 h, fixed, dehydrated through ethanol and hexamethyldisilazane and imaged. *S. alvi* biofilms were grown in BHI media with 0 μg/mL black carbon, 100 μg/mL black carbon or pre-treated media. Another condition, 100 μg/mL quartz, was used as a control to determine if particles of a similar size to black carbon, but chemically inert, affected *S. alvi* biofilm structure.

In all conditions, higher magnification images show hair-like projections (Fig. 2B, C) and black carbon can be seen incorporated into the biofilm structure in close proximity to *S. alvi* cells (Fig. 2C). Pre-treated and quartz-treated biofilms (Supplementary Fig. 5) appear thinner, similar to the 0 μg/mL black carbon treated samples (Fig. 2B, D). The control biofilms showed a relatively flat surface whereas the topography of the black carbon treated biofilms were altered (Fig. 2C, E), black carbon treated biofilms were thicker, more irregular, with larger protrusions extending from the biofilm surface.

**Fig. 3:**
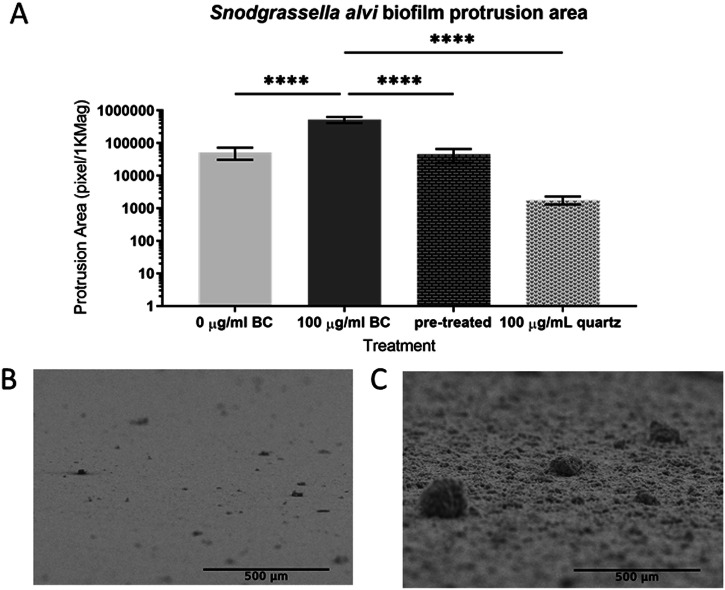
Black carbon particles change *Snodgrassella alvi* biofilm architecture. *S. alvi* wkB2 biofilms were grown for 24 h in BHI with 0 μg/mL black carbon (BC), 100 μg/mL black carbon (BC), 100 μg/mL quartz or in black carbon (100 μg/mL) pre-treated BHI (pre-treated). Biofilms were processed and protrusions imaged with scanning electron microscopy. **A** *S. alvi* biofilm protrusion areas were normalised to 1000x magnification and analysed with a one-way ANOVA and Tukey’s multiple comparisons test, significant differences (****p < 0.0001) are shown, *n* = 6 images for each condition. Representative scanning electron microscopy images of *S. alvi* biofilm protrusions treated with 0 μg/mL black carbon (**B**) or 100 μg/mL black carbon (**C**).

To quantify these changes in biofilm structure, mounted biofilms were tilted 90° to image the two-dimensional area of biofilm protrusions from the surface (Fig. 3A–C), protrusion areas were quantified with ImageJ. *S. alvi* biofilms treated with 100 μg/mL black carbon (Fig. 3C) had significantly larger protrusion areas in comparison to all other treatments (*F*_(3, 20)_ = 18.48, 0 μg/mL black carbon *p* < 0.0001, pre-treated *p* < 0.0001, 100 μg/mL quartz *p* < 0.0001) (Fig. 3A). There were no other statistically significant differences between any other treatments. This provides evidence that black carbon particles, but not chemically inert quartz particles, increase *S. alvi* protrusions changing *S. alvi* biofilm structure.

### The effect of black carbon on *Snodgrassella alvi* gene transcription

Previous work showed that exposure of *S. aureus* to black carbon particles induced an adaptive response and changed *S. aureus* global gene transcription^22^. To determine whether black carbon changes *S. alvi* behaviour through altered gene transcription, RNA was extracted from *S. alvi* exponential planktonic growth and *S. alvi* 24-h biofilms. Little is known about the genes involved in biofilm formation in *S. alvi* therefore the transcription of two genes potentially involved in biofilm structure (*pilD*) and regulation (*lysR*) was assessed with qPCR. The *pilD* gene encodes a prepilin peptidase; an essential gene for *S. alvi* adherence, colonisation and biofilm formation in vivo and in vitro^42^. In other bacterial species the transcriptional activator *lysR* is important for regulating biofilm formation, the oxidative stress response and colonisation^55–57^. *S. alvi* possesses a homologue of *lysR*^17^ but its function is unconfirmed.

**Fig. 4:**
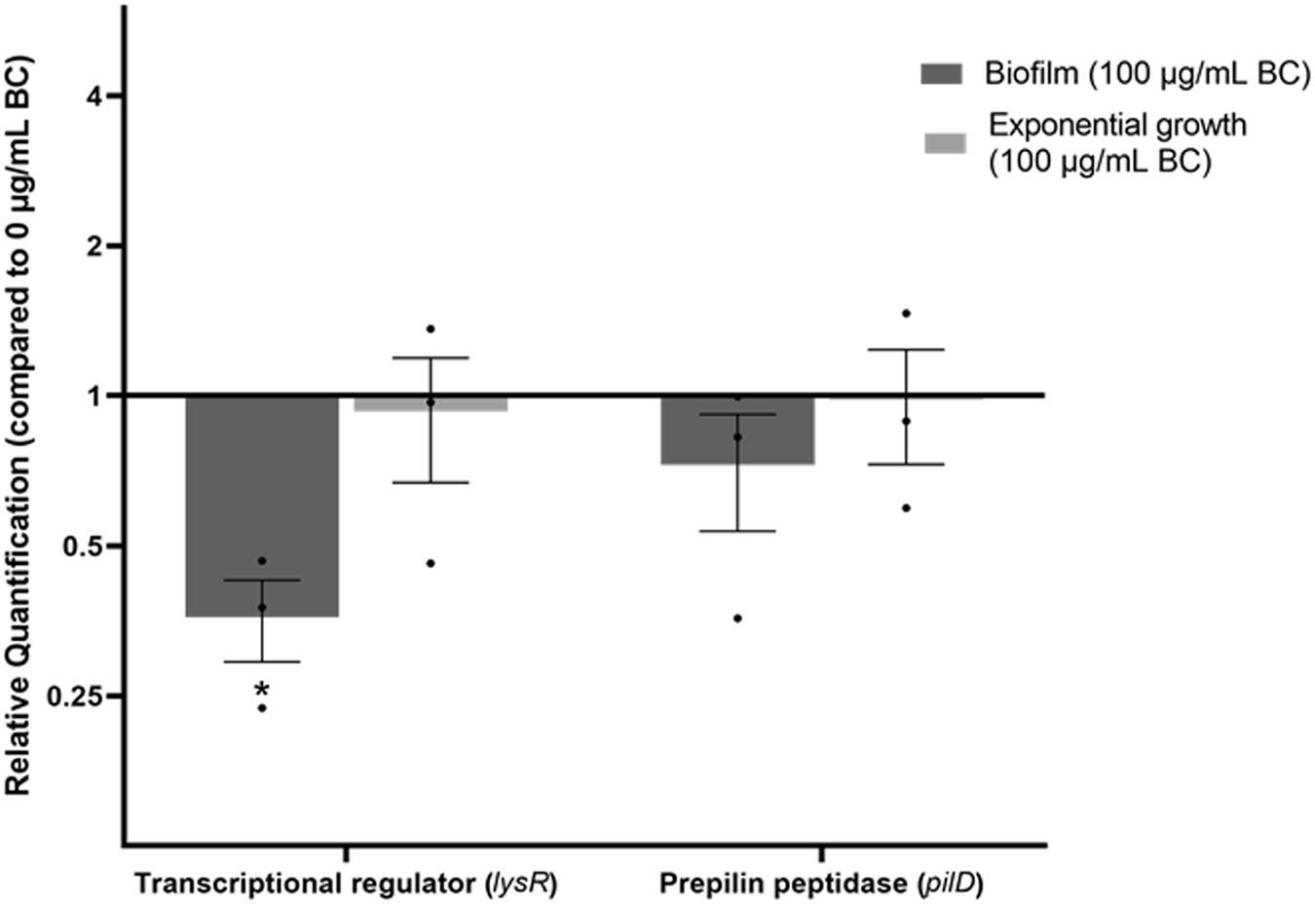
The effect of black carbon on *Snodgrassella alvi* gene transcription. Relative fold change of *S. alvi* wkB2 *lysR* and *pilD* gene transcription when grown exponentially or in a biofilm with 100 μg/mL black carbon (BC). Relative quantification is the fold change in transcription relative to 0 μg/mL black carbon. The endogenous control, *16S* rRNA, was used as its transcription level did not change between control and black carbon treatment. Biological replicates *n* = 3, error bars represent standard error of the mean. A two-way ANOVA was performed on each sample type (exponential planktonic growth and biofilm) with Fisher’s LSD correction, significant differences (*p < 0.05) are indicated.

*S. alvi* biofilms formed in the presence of black carbon had no significant change in *pilD* transcription (*F*_(1, 8)_ = 7.09, *p* > 0.05) but showed a significant decrease in *lysR* transcription (*p* < 0.05) (Fig. 4). These changes were not observed in exponential planktonically grown cells (Fig. 4) (*F*_(1, 8)_ = 0.11, *p* > 0.05).

### Black carbon perturbs the bumblebee gut microbiome at a concentration non-toxic to the host

The gut microbiome is critical to the health of the bumblebee therefore the impact of black carbon exposure on the established bumblebee gut microbiome in vivo and subsequent changes to bee activity and survival were investigated, in lab-reared adult *B. terrestris* in a controlled environment.

**Fig. 5:**
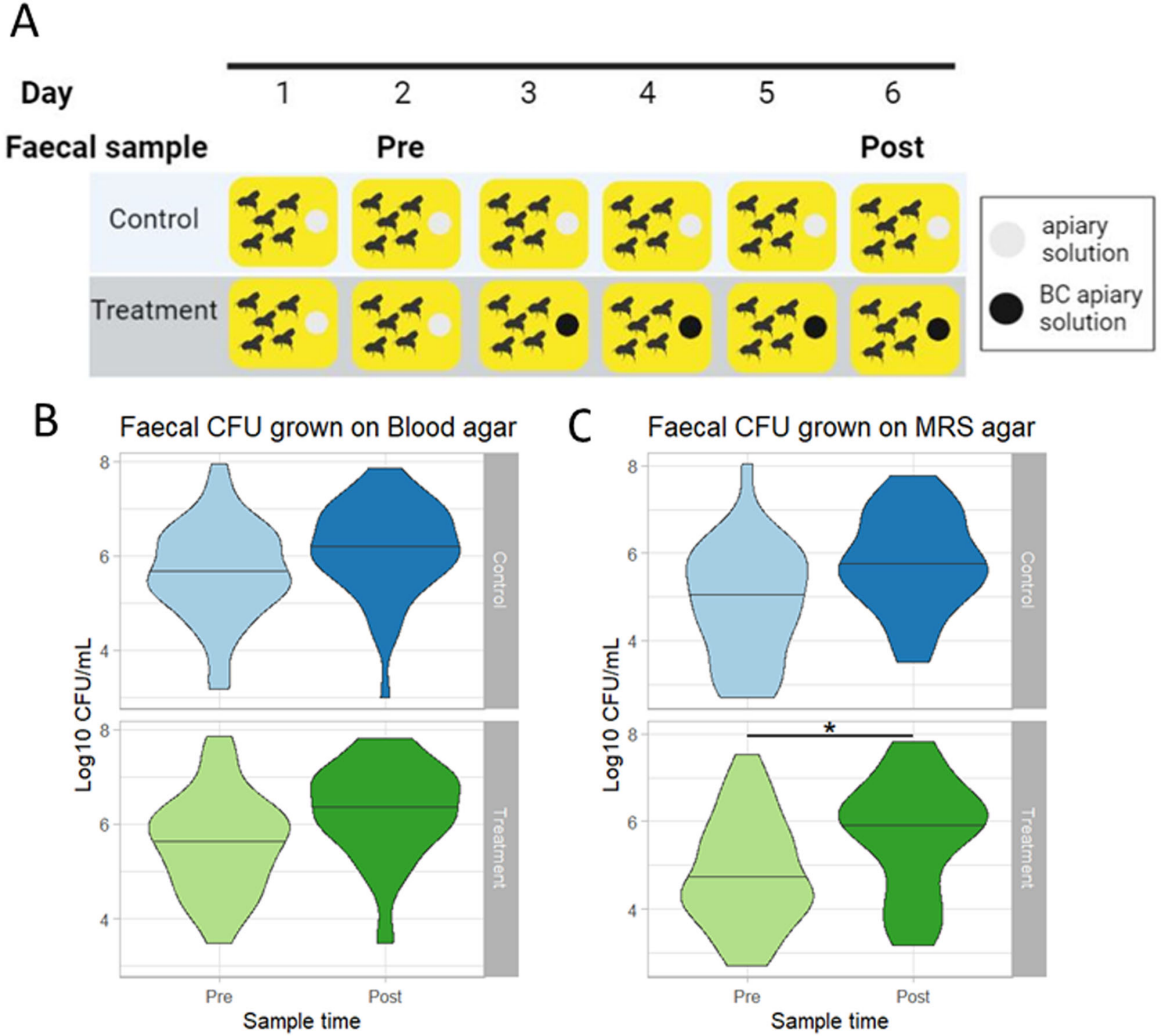
The effect of black carbon on adult *Bombus terrestris* viable gut bacteria. **A** Experimental setup showing feeding patterns with apiary solution or black carbon (BC) laced apiary solution (0.495 mg/g) and sample collection across a daily period (created with Biorender.com). **B**, **C** *B. terrestris* faecal CFU per experimental group (control *n* = 42; black carbon treatment *n* = 52), lines represent median. Faecal samples were grown on blood agar (**B**) or MRS agar (**C**) and incubated for 72 h in microaerobic conditions (34 °C, CO_2_: 10%, O2:5%, N2:85%). CFU results from experimental groups were analysed with a general linear model and a two-way ANOVA with multivariate *p* value adjustment, significant differences (*p < 0.05) are indicated.

Age controlled adult worker bees with a fully formed, mature gut microbiome (7 ± 1 day old) were moved into experimental boxes and designated either treatment or control group. Both experimental groups were fed apiary solution for two days (Pre), after which fresh apiary solution was provided alone (control) or with 0.495 mg/g black carbon (treatment) for the remaining four days of the experiment (Post) (Fig. 5A).

To determine if black carbon influenced bee behaviour, bee active time was recorded for control and treatment experimental groups Pre and Post treatment. Bees were randomly selected per experimental box and their activity was recorded for 150 s. There was no significant difference in active time Pre and Post for either experimental group (Supplementary Fig. 6) (control W = 100.5, *p* > 0.05; treatment W = 86.5, *p* > 0.05). The effect of black carbon treatment on bee mortality rates was assessed by monitoring bee survival periodically throughout the experiment. Control and treatment groups had no significant difference (W = 14.5, *p* > 0.05) in mortality with the majority of bees from each experimental group surviving until the end of the experiment (Supplementary Fig. 7). As bees treated with black carbon did not have any significant differences in survival or activity levels compared to controls the dose of black carbon used in these experiments does not appear to be overtly toxic to the host.

To determine the effects of black carbon on gut microbiome composition, faecal samples were taken^58^ at two timepoints Pre (day two) and Post black carbon exposure (day six). Faecal samples were used to monitor the gut microbiome composition over time. Part of each sample was cultured to quantify viable bacteria; the remaining samples were pooled by experimental box and DNA extracted for 16S rRNA amplicon sequencing.

For quantification of viable microaerophilic bacteria, a complex non-selective medium (blood agar) (Fig. 5B) and a lactobacilli-selective culture medium (MRS agar)^59^ (Fig. 5C) were used to culture bee faecal samples taken at Pre and Post timepoints from control bees and those treated with black carbon.

Bacterial viability scores were analysed with a negative binomial regression, finding a significant interaction between experimental group, sample time and media (Negative binomial regression: χ^2^(1) = 352.79, *p* < 0.05) (Supplementary Table 1). The bee gut microbiome cultured on MRS, showed no significant difference (*p* > 0.05) in the control group bacterial viability. The only significant post hoc result (Supplementary Table 2) was a significant increase in MRS viability Post black carbon treatment compared to Pre-treatment (multivariate *p* value adjustment: z score: 2.971, *p* < 0.05) (Fig. 5C). Black carbon exposure significantly increased MRS-culturable bacteria changing the adult *B. terrestris* gut microbiome.

To determine which members of the bee gut microbiome are altered with black carbon treatment, the remaining faecal samples were pooled by experiment box (control *n* = 14 boxes; black carbon treatment *n* = 16 boxes) and DNA extracted. Total 16S rRNA gene copy number was quantified by qPCR, finding no significant difference (*F*_(3, 18)_ = 0.86, *p* > 0.05) in absolute 16S abundance for any experimental group (Supplementary Fig. 8). DNA was 16S rRNA amplicon sequenced, all boxes were sampled twice, before (Pre) and after (Post) treatment, only samples from boxes that passed sequencing quality control for both Pre and Post timepoints were used in further analyses to compare the diversity and relative abundance of gut bacteria (control *n* = 4 boxes; black carbon treatment *n* = 7 boxes). Amplicon sequence variants (ASV) were identified and assigned taxa using the BEExact database^60^. The relative abundance of sample genera was plotted for each sample.

**Fig. 6:**
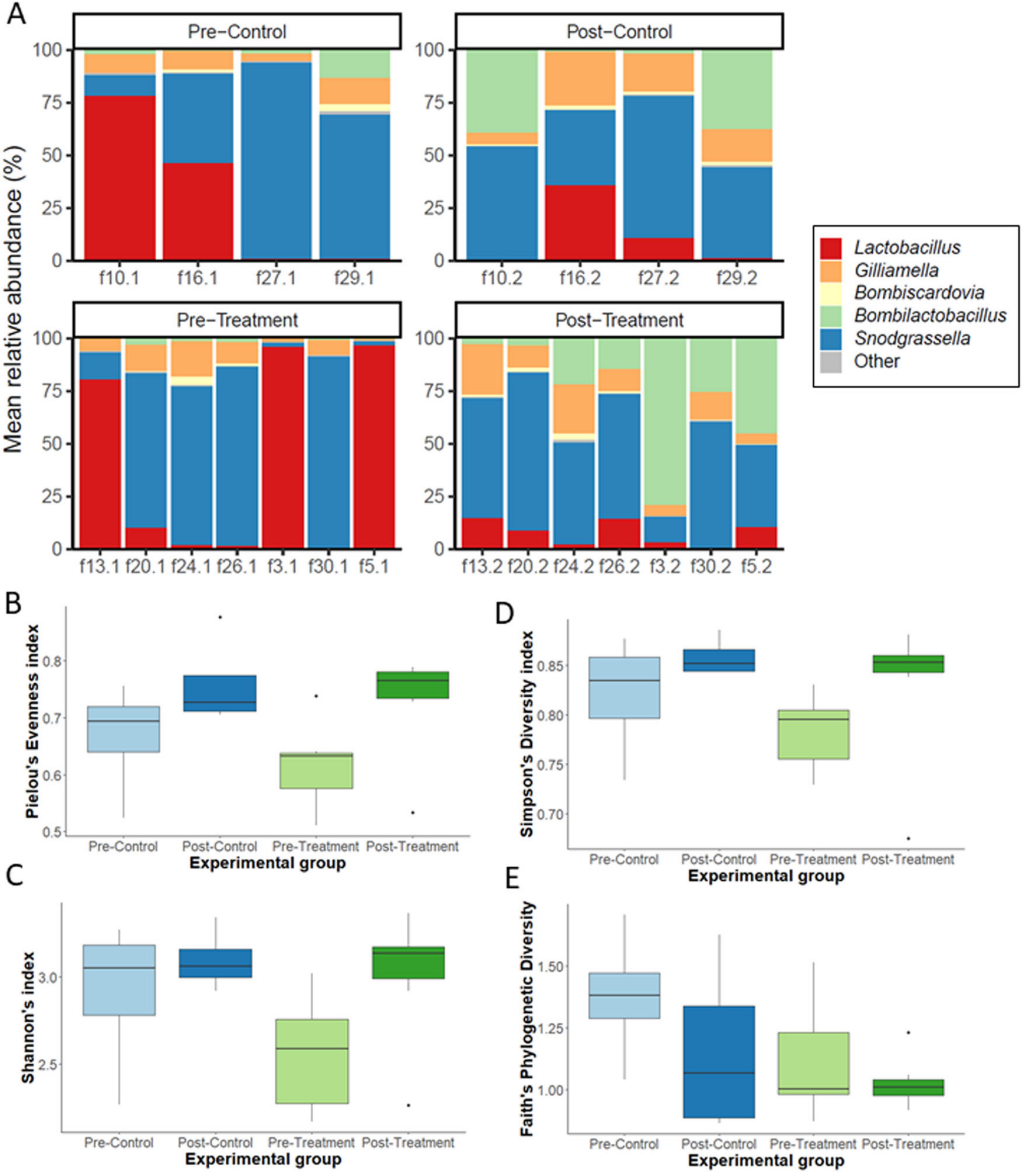
The effect of black carbon on the composition and diversity of adult *Bombus terrestris* gut microbiome. **A** Mean relative abundance of genera per sample box, displayed by experimental group (control *n* = 4, black carbon treatment *n* = 7). 16S rRNA amplicon sequences were assigned taxa using the BEExact database in Qiime2 and visualised with the phyloseq package in R. Taxa that were <1% of the total relative abundance are categorised in ‘Other’. Alpha diversity metrics Pielou’s evenness index (**B**), Shannon’s index (**C**), Simpsons’s diversity index (**D**) and Faith’s phylogenetic diversity (**E**) were calculated for each experimental group. Kruskal-Wallis pairwise tests, and False Discovery Rate corrections were conducted for each alpha diversity metric and found no significant differences at the 0.05 level between Pre and Post control and black carbon treatment groups.

As faecal samples were collected from the same bees at two timepoints (Pre and Post), the microbiome composition of samples can be compared between these experimental groups (Fig. 6A) to determine the changes to the gut microbiome over time (control) and the effects of black carbon (treatment). Both experimental groups had comparable compositions and relative abundances at the Pre timepoint and no sample ASVs matched with negative controls. Experimental groups were dominated by five genera *Lactobacillus*, *Gilliamella*, *Bombiscardovia, Bombilactobacillus* and *Snodgrassella* (Fig. 6A, Supplementary Fig. 9) which are all core members of the bumblebee gut microbiome^32,38^. Genus *Snodgrassella* was the most abundant overall, with other core taxa occupying varying amounts of the remaining relative abundance.

Alpha and beta diversity analyses were conducted to determine diversity within and between experimental groups. Higher alpha diversity values indicate more diversity and evenly distributed genera. There were increases in the alpha diversity metrics Pielou’s evenness index, Shannon’s index and Simpson’s index, Post black carbon treatment compared to Pre-treatment (Fig. 6B–E), yet these differences were not significant (*q* > 0.05). Bray Curtis PCoA and NMDS plots (Supplementary Fig. 10) showed some similarity within experimental groups, however these differences were not significant (*p* > 0.05) with PERMANOVA analyses.

Absolute abundance values were calculated, adjusting for 16S rRNA copy number (Supplementary Fig. 11), and the statistical test, analysis of compositions of microbiomes with bias correction (ANCOM-BC)^61^, was used to identify differentially abundant genera within experimental groups. After black carbon treatment core bacterial taxa *Bombilactobacillus* in the Post-treatment group was significantly differentially abundant (*q* < 0.001) compared to Pre-treatment (Table 1). There were no differentially abundant core bacteria between the Pre control and Post control experimental groups. Black carbon treatment significantly increased the abundance of core symbiont *Bombilactobacillus* changing the adult *B. terrestris* gut microbiome. These findings provide evidence that, even at a sublethal dose, exposure of adult *B. terrestris* to black carbon particulates significantly increased viable bacteria on MRS agar and 16S absolute abundance of beneficial bacteria *Bombilactobacillus* in Post treated bumblebees compared to Pre-treated. Importantly, the subtle, yet distinct changes in our data show that low levels of non-toxic particulates can cause changes to microbiomes.

**Table 1:** Differential abundance ANCOM-BC scores of core bacterial taxa, analysed within experimental group

	*Bombilactobacillus*		*Gilliamella*		*Lactobacillus*		*Snodgrassella*		*Bombiscardovia*	
	Control	Treatment	Control	Treatment	Control	Treatment	Control	Treatment	Control	Treatment
beta coefficients	−0.72822	−2.23866	−0.28318	−0.09059	0.961995	1.329129	0.158642	−0.09387	−0.18469	−0.10679
Standard error	1.215786	0.636633	0.88478	0.431075	1.897953	1.120167	1.043772	0.670423	0.796415	0.64193
W statistic	−0.59897	−3.51641	−0.32006	−0.21016	0.506859	1.186545	0.151989	−0.14001	−0.2319	−0.16636
*p* value	0.549191	0.000437	0.748926	0.833545	0.612254	0.235407	0.879196	0.888652	0.816617	0.867876
q value	0.549191	0.005249	0.748926	1	0.612254	1	0.879196	1	0.816617	1
Differential abundance	FALSE	TRUE	FALSE	FALSE	FALSE	FALSE	FALSE	FALSE	FALSE	FALSE

## Discussion

In this study, we have shown that black carbon particulates significantly alter biofilm formation and gene transcription of key bee gut commensal *Snodgrassella alvi* in vitro. Exposure to black carbon increased MRS-culturable gut bacteria in adult *Bombus terrestris* and increased the abundance of *Bombilactobacillus* disrupting the *B. terrestris* gut microbiome composition. Our data provides the first evidence that a chemically defined, single type of particulate pollution alters both the behaviour of bees’ commensal bacteria in vitro and their gut microbiome composition in vivo. The black carbon used in this study is 99% carbon, and only contains trace amounts of toxic pollutants such as heavy metals and no polycyclic aromatic hydrocarbons, yet there is a reproducible effect on bacterial phenotypes. These findings provide evidence that non-toxic particulate air pollution has significant effects on beneficial commensal bacteria which may contribute to air pollutions’ detrimental effects on bee health.

*Snodgrassella alvi* is a key, beneficial member of the core gut microbiome in honeybees and bumblebees^32^ and is able to persist in an otherwise sterile gut without the presence of other core gut microbes^40^. *S. alvi* predominantly colonises the ilea forming a biofilm structure on the ileum epithelium^36,41^. We found that treatment with black carbon changed *S. alvi* wkB2 biofilms, significantly increasing initial adhesion to an abiotic surface in vitro and changing early biofilm formation. Our data show an increase in the number of viable biofilm cells at 2 h, 8 h, and 24 h but no change at 48 h and no overall effect of black carbon on *S. alvi* growth. Black carbon particulates were not observed, incorporated with adhering cells at 2 h and therefore could not provide a physical support for biofilm formation at this timepoint.

Transcriptional analysis showed that exposure to black carbon significantly decreased the transcription of the gene *lysR* in *S. alvi* biofilm cells but not in planktonic cells. As *lysR* is a regulator with diverse functions, the change in transcription with black carbon treatment potentially has further effects on *S. alvi* functions and internal metabolism. LysR homologues in other bacteria, including the closely related species *Neisseria gonorrhoeae*, are involved in the regulation of colonisation, the oxidative stress response and biofilm formation^55–57^. The transcription of *pilD* in *S. alvi* 24-h biofilms was also tested, finding no difference in transcription with black carbon treatment. As black carbon significantly increases *S. alvi* early biofilm viability, the transcription of biofilm related genes may also be altered by black carbon in a time related manner. Therefore, while the function of the *S. alvi lysR* gene has not been tested, the significant alteration in *lysR* transcription in black carbon treated *S. alvi* biofilms suggests that *lysR* may play a role in *S. alvi* biofilm regulation. These data provide evidence of a genetic basis for the phenotypic changes of black carbon on *S. alvi* biofilms, consistent with other bacteria that show adaptive genetic responses to black carbon and urban particulate pollutants^21,22^.

Scanning electron microscopy images showed that the presence of black carbon dramatically alters the structure of *S. alvi* biofilms. Black carbon particulates were observed incorporated into the 24-h biofilm structure and there was a significant increase in the size of biofilm protrusions, which could destabilise the biofilm when under shear stresses. Significant changes to biofilm protrusion structures were not observed in biofilms exposed to black carbon pre-treated media or chemically inert quartz particles. Particulate pollutants alter biofilm structures in other bacterial species treated with black carbon^19^ and different particulate pollutants (urban dust and fine particulate matter)^20,21^. These studies also found particulate pollutants incorporated into the biofilm^20,21^ and showed similar protrusion structures in black carbon treated *S. aureus*^19^. The mechanisms involved were not established.

We propose that black carbon particulates alter *S. alvi* biofilm formation through physical interaction with the bacteria and induction of a genetic adaptive response. Our data shows that physical interaction between *S. alvi* and black carbon particulates >0.2 μM, play a critical role in later stages of biofilm formation because the presence of black carbon particulates is required to change the biofilm structure. There is no change in biofilm formation when *S. alvi* are grown in medium pre-treated with black carbon in the absence of these particulates. Notably, the change in biofilm structure is not only through particle-bacteria physical interactions supporting the biofilm structure because *S. alvi* initial adhesion is induced at 2 h, before black carbon particulates are detected in the adherent biofilm cells. Additionally, black carbon alters the transcription of the *lysR* gene, a potential regulator of *S. alvi* biofilm formation.

Importantly, increased early biofilm formation along with larger biofilm protrusions of prominent core gut bacteria *S. alvi* in vitro suggests that spatial organisation of microbes in the gut, such as *S. alvi* on the ileal epithelium, could be disrupted with exposure to black carbon in vivo. Indeed, our in vivo results show subtle but longer-term changes to the mature bee gut microbiome with black carbon exposure. Future investigations could determine if disrupting the gut microbiome organisation may in turn alter the abundance of other core gut microbes.

To study the impact of black carbon on *B. terrestris* gut microbiome, age controlled adult worker bees, with a fully formed mature gut microbiome, were subjected to an exposure relevant concentration of black carbon through consumption in their apiary solution. *B. terrestris* exposed to black carbon did not have significantly different survival compared to the control group. Overall, the majority of *B. terrestris* from each experimental group survived until the end of the experiment, which would be expected at their age^62^. Additionally, black carbon had no significant effect on *B. terrestris* activity levels compared to controls. Therefore, this concentration of black carbon was not overtly toxic to *B. terrestris* over the experimental period.

There is evidence of the impact of air pollution on wild bee survival showing that air pollution could be having an under-appreciated impact on bee health. Previous work on solitary and social bee species report an association between high environmental air pollution levels and a lower abundance of wild bees^52,63,64^.

Previous studies have also shown pollution-mediated changes to honeybee foraging behaviour, *A. dorsata* floral visitation rates were significantly decreased when exposed to high air pollution levels^52^. For in vivo experiments, *B. terrestris* exposed to toxic concentrations of diesel exhaust particles had lower fat body content and a reduction in their food consumption, providing evidence that exposure to particulates can negatively impact bees^65^. Furthermore pollution exposure impairs *Apis mellifera* cognitive processes (olfactory learning and memory) that are important for foraging^66,67^. Bumblebees are primitively eusocial insects and therefore any changes to foraging (and subsequent pollination) would have secondary effects on colony success, pollination rates and ultimately our food production.

Our data demonstrates that, although the concentration of black carbon used in these experiments did not overtly affect bee health, black carbon significantly increases viable microaerophilic *B. terrestris* gut bacteria. *B. terrestris* had significantly more MRS culturable bacteria (such as *Bombilactobacillus* and *Lactobacillus* Firm-5) Post black carbon treatment compared to Pre treatment. Furthermore, 16S rRNA amplicon sequencing of the faecal samples revealed that black carbon significantly increases the abundance of beneficial core phylotype *Bombilactobacillus*, changing the composition of *B. terrestris* mature gut microbiome. Importantly, *B. terrestris* were exposed to black carbon after obtaining their fully formed, mature gut microbiome, demonstrating that black carbon treatment disrupts the established bee gut microbiome.

Previous work investigating the impact of the heavy metal selenate on *Bombus impatiens*^48^ and diesel exhaust particles on *B. terrestris*^18^ also saw alterations in the abundance of core gut symbionts in bees treated with these pollutants. In comparison to control bees, both studies found a lower abundance of *Snodgrassella* and *Lactobacillus bombicola* (*Lactobacillus* Firm-5) and an alteration in *Gilliamella* abundance in pollution treated bees. Changes in *Lactobacillus apis* (*Lactobacillus* Firm-5) and *Bombiscardova* abundance have also been reported with pollution treatment^18^. We found that bees exposed to black carbon pollution had a significantly higher abundance of *Bombilactobacillus* (*Lactobacillus* Firm-4), which was not found in previous work. Although, there was an increase in the abundance of core microbes *Snodgrassella* and *Gilliamella*, after exposure to black carbon, significant changes in these symbionts and other core microbes were not observed in our data. Consequently, these studies highlight the importance of fully establishing the impact of different pollutants on insect pollinator health because different pollutants have distinct, specific effects on the abundance of core bee gut microbes. *B. terrestris* exposed to high concentrations of black carbon (20 mg/g) had no effect on bee survival^65^ whereas higher concentrations of selenate and diesel exhaust particulates have a toxic effect on bees, significantly decreasing their survival^18,48^. Therefore, we have shown that exposure to non-toxic particulate pollutants can have significant effects.

*Bombilactobacillus* (*Lactobacillus* Firm-4) is a core bee gut bacteria that influences hormonal signalling and is involved in the metabolism of pollen, short chain fatty acids and glycosides^33,34^. Black carbon exposure altered the abundance of core symbiont *Bombilactobacillus* in *B. terrestris*, consequently, this may affect the functionality of the bee gut microbiome such as the ability to utilise a variety of different food sources. Our findings show that short term ingestion of black carbon has distinct, significant effects on the composition of the established, mature *B. terrestris* core bacterial community. These results could describe a key mechanism whereby air pollution affects bee physiology and may have serious implications for host health and pollination.

Air pollution particulate matter has a pervasive presence in our atmosphere and accumulates on bee’s bodies and in their food stores. Yet, there is limited knowledge on how particulates affect bee health and to what extent. In this study, we focused our investigations on the core bee gut microbiome as its composition is common to major pollinators (honeybees and bumblebees) and is strongly linked to bee health^32,48,68^. We have shown that a chemically defined major particulate air pollutant black carbon, has significant effects on core bacteria, differentially altering the composition of *Bombus terrestris* beneficial core gut microbiome. These findings demonstrate the importance of establishing the full impact of air pollution directly on bacteria within beneficial microbial communities, such as the bee gut microbiome, and the subsequent influence on the health of our essential insect pollinators. Together our data show that particulate air pollutants are an underexplored risk for the health and balance of environmental microbes and their important natural ecosystems. Policy discussions concerning air pollution and emission reduction would benefit from considering agriculturally important pollinators for the future of our global food security.

## Methods

### Black carbon and quartz particles

Black carbon (BC) (Sigma-Aldrich 699632) powder was suspended in sterile ddH_2_O^19,22^ to make a stock solution at 2 mg/mL. The black carbon particulates consist of over 99% carbon, with a particle size of <0.5 µm and contain <500 ppm of trace metals. Black carbon will be used as a model particulate because it is chemically defined, not toxic at the concentrations used and available in a consistent and stable form required for the reductionist approach needed for reproducible and comparative mechanistic studies. Quartz particulates, size 3.5–0.35 µm, (Distrilab BCR66) were suspended in sterile ddH_2_O. Quartz is chemically stable and does not react with most substances even at high temperatures and is therefore used as a chemically inert particle control in this study. All particulate stock solutions were homogenised immediately before use in experiments, to maintain accuracy and mitigate for any particulate settling. Particulate sizes used in this study are within the range of environmental particulate pollutant sizes found to accumulate on wild bees (10–0.02 µm)^52,69^.

### Bacterial growth conditions

The bee gut commensal type strain *Snodgrassella alvi* wkB2 (DSMZ, DSM 104735), isolated from *Apis mellifera*^70^, was used in this study. *S. alvi* was stored at −80 °C and cultivated from frozen glycerol stocks on blood agar (Oxoid). Unless otherwise stated, bacteria were grown using blood agar (Oxoid) supplemented with 5% horse blood (Oxoid), Brain Heart Infusion broth (BHI, Oxoid) and incubated statically in 5% CO_2_ and 37 °C conditions.

BHI medium pre-treated with black carbon was made by supplementing sterile BHI media with 100 µg/mL black carbon, vortexed for 3 s to suspend the particulates and incubated at 37 °C overnight. Media was centrifuged for 10 min at 3220 × g 22 °C to pellet the black carbon particles and supernatant was taken, leaving some media behind to avoid the pellet and additionally passed through a 0.2 µm filter.

### *Snodgrassella alvi* biofilm assay

The effect of black carbon on *S. alvi* wkB2 biofilm formation was tested in vitro by quantifying the viability of different fractional components of the biofilm. Overnight cultures of *S. alvi* wkB2 were set to an optical density of 0.02 at 600 nm in BHI, 3 mL was aliquoted into a 12 well plate with or without 100 µg/mL of black carbon and incubated in 5% CO_2_ at 37 °C for 2 h, 8 h, 24 h or 48 h. A previous published protocol was followed^19^ except for the use of BHI for washes and dilutions. Fractions were vortexed for 10 s, samples were serially diluted in BHI and plated onto blood agar plates to count colony forming units (CFU). To image biofilms, samples were set up and incubated as above, supernatant was removed, and the biofilm stained with crystal violet for 5 min. The crystal violet stain was removed, biofilms were washed with ddH_2_O and imaged using Immunospot® (Cellular Technology Limited).

### Scanning electron microscopy

To establish what structural biofilm changes occur in the presence of black carbon, bacterial cultures were inoculated onto sterile plastic coverslips (Agar Scientific AGL4193) within 12 well plates, grown with 0 μg/mL black carbon, 100 μg/mL black carbon, 100 μg/mL quartz or pre-treated media and samples were incubated at 5% CO_2_ and 37 °C for 24 h. The 100 μg/mL quartz condition was used as a control to determine if particles of a similar size to black carbon, but chemically inert, affected *S. alvi* biofilm structure. After incubation, coverslips were fixed with 2.5% glutaraldehyde in 0.1 M Sörensens buffer for 2 h at room temperature^71^. After fixing, biofilms were washed in PBS, ddH_2_O and dehydrated in an ethanol series, then a graded series of ethanol:hexamethyldisilazane mixtures. Samples were air dried overnight, mounted onto pin stubs, gold coated and viewed with a Hitachi S-3000H. To image biofilm structures, stubs were tilted 90° and biofilm protrusions were imaged in cross sections by random selection, framing, focussing and capturing, protrusion areas of these captured images were measured from the surface of the coverslip and analysed using the image threshold function in ImageJ^72^.

### RNA extraction

RNA was collected from *S. alvi* planktonic growth samples and *S. alvi* biofilm samples. For planktonic growth, *S. alvi* was grown to exponential phase (OD_600 nm_ = 0.08) in BHI with and without 100 µg/mL black carbon. RNA was stabilised with the addition of one fifth the culture volume of 1:19 Phenol:Ethanol and incubated on ice for ~25 min. The culture was centrifuged at 4000 rpm (3220 × g), 4 °C for 5 min to pellet the cells. The supernatant was discarded, and the pelleted cells were stored at −80 °C.

To prepare *S. alvi* biofilm samples for RNA extraction, *S. alvi* biofilms were grown in BHI with and without 100 µg/mL black carbon and incubated for 24 h as above. Supernatant and wash fractions were removed and discarded, RNA was stabilised with the addition of 1 mL RNAlater (ThermoFisher) and incubated for 5 min at room temperature. The biofilm fraction was transferred to a microcentrifuge tube, centrifuged (11400 × g) for 5 min. The supernatant was discarded, and the pelleted cells were stored at −80 °C.

RNA was extracted following a similar protocol as previous work^22^, except frozen pellets were thawed and lysed in 200 µL of Tris-EDTA buffer containing 20 µL of lysosyme (1 mg/mL) and incubated at 37 °C for 15 min prior to being mechanically disrupted using Lysing Matrix B tubes and MP Biomedicals FastPrep-24 Classic instrument (MP Biosystems). RNA was extracted from the supernatant using a Direct-zol RNA Miniprep kit (Zymogen) following the manufacturer’s instructions and samples were further treated as described previously^22^.

### Quantitative reverse transcriptase PCR (qRT-PCR)

Total RNA was converted to cDNA using Superscript IV VILO Master Mix reverse transcriptase (Invitrogen) and 1 ng of cDNA was used for each qPCR reaction. Reactions (10 µL) were carried out in triplicate using SYBR Green Master mix in a 7300 Fast System (Applied Biosystems) following manufactures instructions. Relative gene expression for each sample (primer details Supplementary Table 3) were normalised to the expression of endogenous control 16S rRNA gene and analysed relative to *S. alvi* cultured without black carbon. Relative quantification was calculated using ∆∆Ct analysis^73^.

### Bumblebee rearing and husbandry

The impact of black carbon exposure on the bumblebee gut microbiome and subsequent changes to bee activity and survival were investigated in lab-reared adult *B. terrestris*. Four *Bombus terrestris audax* colonies, were ordered from Biobest (Westerlo, Belgium) in January 2019. Colonies were reared in constant red-light conditions at 28 °C and 60% humidity. They were fed 60% v/v apiary solution and water (Meliose-Roquette, France) and pollen (Percie du sert, France) *ad libitum*. Callow workers, less than 24 h old, were caught, labelled on the thorax with a marker, their number recorded and returned to the colony. Once they had obtained their mature, adult gut microbiome (7 ± 1 day old^46^), marked bees were placed into rearing boxes of four to six individuals and designated either treatment (*n* = 16 boxes) or control (*n* = 14 boxes) experimental group. Experimental groups were reared in constant red-light conditions at 28 °C and 60% humidity and fed pollen and apiary solution and water (70% v/v) *ad libitum*.

### Experimental setup, black carbon exposure and sampling

Both experimental groups were fed apiary solution and water (70% v/v) for two days. After faecal sample collection on day two the control group was provided fresh apiary solution, and the treatment group was given apiary solution containing black carbon (0.495 mg/g black carbon as calculated in Supplementary Fig. 12) for the remaining four days of the experiment.

The dose of black carbon used was calculated (Supplementary Fig. 12) using published exposures to humans^54^ but accounted for bee dimensions, average daily consumption of apiary solution^74^ and bee weight^75^. The concentrations used reflect those used in previous studies and bee studies using apiary syrup and environmental pollutants that were adjusted to environmental exposure levels^18,48,76,77^.

### Bee survival and activity monitoring

To determine if black carbon influenced bee behaviour, activity was recorded from one bee, selected at random (using a random number generator) per experimental box. Twice each day bees were randomly selected per experimental box and their active time was recorded for 150 s. Survival was measured periodically throughout the experiment to monitor host mortality with black carbon exposure.

### Faecal sampling, DNA extraction and total 16S rRNA copy number

To determine the effects of black carbon on gut microbiome composition over time, faecal samples were taken from anaesthetised bees at two timepoints. On experimental days Pre (day two) and Post black carbon exposure (day six) bees were individually caught, placed in ventilated universal tubes and anaesthetised on ice for ~20 min prior to faecal sample collection^58^. Fresh faecal samples were diluted in 100 µL of sodium phosphate buffer from the FastDNA SPIN kit for soil^78^ and briefly vortexed. From each sample, 20 µL was taken for bacterial culture and the remaining sample was frozen at −20 °C. For bacterial culture, each sample was serially diluted, plated onto De Man, Rogosa and Sharpe (MRS, Oxoid) agar^59^ and blood agar and incubated in microaerobic (34 °C, 10% CO_2_, 85% N_2_, 5% O_2_) conditions for 72 h. After incubation, viable counts were recorded to calculate CFU.

The remaining faecal samples were thawed, pooled according to experimental box and processed with the FastDNA SPIN kit for soil^78^. Samples were paired-end sequenced on an Illumina 1.9 platform. The V3-V4 region of the 16S rRNA gene was amplified with primers 341 F (5-CCTAYGGGRBGCASCAG-3) and 806 R (5-GGACTACNNGGGTATCTAAT-3). Negative controls for the DNA extraction kit were amplified for the V4 region with primers 515 F (5-GTGCCAGCMGCCGCGGTAA-3) and 806 R (5-GGACTACHVGGGTWTCTAAT-3).

Total 16S rRNA copy number was amplified from all samples using universal bacterial primers (Supplementary Table 3) as in previous studies^68,79^. Reactions (10 µL) were carried out in triplicate using SYBR Green Master mix in a 7300 Fast System (Applied Biosystems), using PCR cycles as described in previous work^68^. Standard curves from the amplification of the cloned target sequence in a pGEM-T vector (Promega) were used to quantify 16S total copy number per sample. Absolute abundance of each bacterial taxa was estimated by multiplying the total number of 16S rRNA genes measured with qPCR (adjusting for rRNA operons per genome) by the percentage of relative abundance per taxa. The number of 16S rRNA operons was determined using the reference genome when available (Supplementary Table 4) or closely related species/genus mean 16S rRNA operon copy number was obtained from the rrnDB (https://rrndb.umms.med.umich.edu/).

### Bioinformatics pipeline

Individual sample quality was determined with FastQC^80^ and summarised with MultiQC v1.9^81^. Illumina paired-end reads were processed in Qiime2 v2020.2^82^. Barcodes and primers were trimmed with DADA2^83^, sequences were denoised, filtered for quality, merged, chimeric sequences were removed and features identified. The bee-associated microbial community database BEExact^60^, was trained for the appropriate 16S region and used to taxonomically assign samples’ amplicon sequence variants^84^. Non-rarefied data was used to calculate relative abundance, absolute abundance was subsequently calculated using these values, adjusting for 16S rRNA copy number. Differential abundance analysis was conducted within experimental groups using absolute abundance values and was carried out in R using ANCOM-BC using the default settings, accounting for sample size and the following parameters: lib_cut = 1000^61^. Rarefaction, filtering and alpha and beta diversity analysis^85,86^ was carried out in Qiime2 and R^87^ utilising phyloseq^88^, ggplot2^89^ and tidyverse^90^ packages.

### Graphical output, statistics and image analysis

Graphs were produced and statistical analysis performed using R statistical software^87^ or GraphPad Prism (version 7.0.4 for Windows, GraphPad Software, San Diego, California USA). Significant difference was determined as *p* < 0.05. ANOVA were used to determine significance as appropriate. Šidák post hoc correction was used for multiple comparison testing between more than two groups, Tukey’s multiple comparisons test was used to correct for comparing all pairs of means. For unpaired data, Kruskal-Wallis with False Discovery Rate post hoc test for multiple comparison testing was used. Scanning electron microscopy images were processed using ImageJ^72^.

## Supplementary information

supplementary material

## Data Availability

Data is freely available on request (https://figshare.le.ac.uk).
