# Supplementary material for: Air pollution modifies colonisation factors in beneficial symbiont Snodgrassella and disrupts the bumblebee gut microbiome
Source: NPJ Biofilms Microbiomes. 2025 Jan 2;11:2. doi: 10.1038/s41522-024-00632-3 (PMC11699285; doi:10.1038/s41522-024-00632-3)
Supplement: Supplementary file 1 — supplementary material [file 41522_2024_632_MOESM1_ESM.pdf]

Supplementary Information

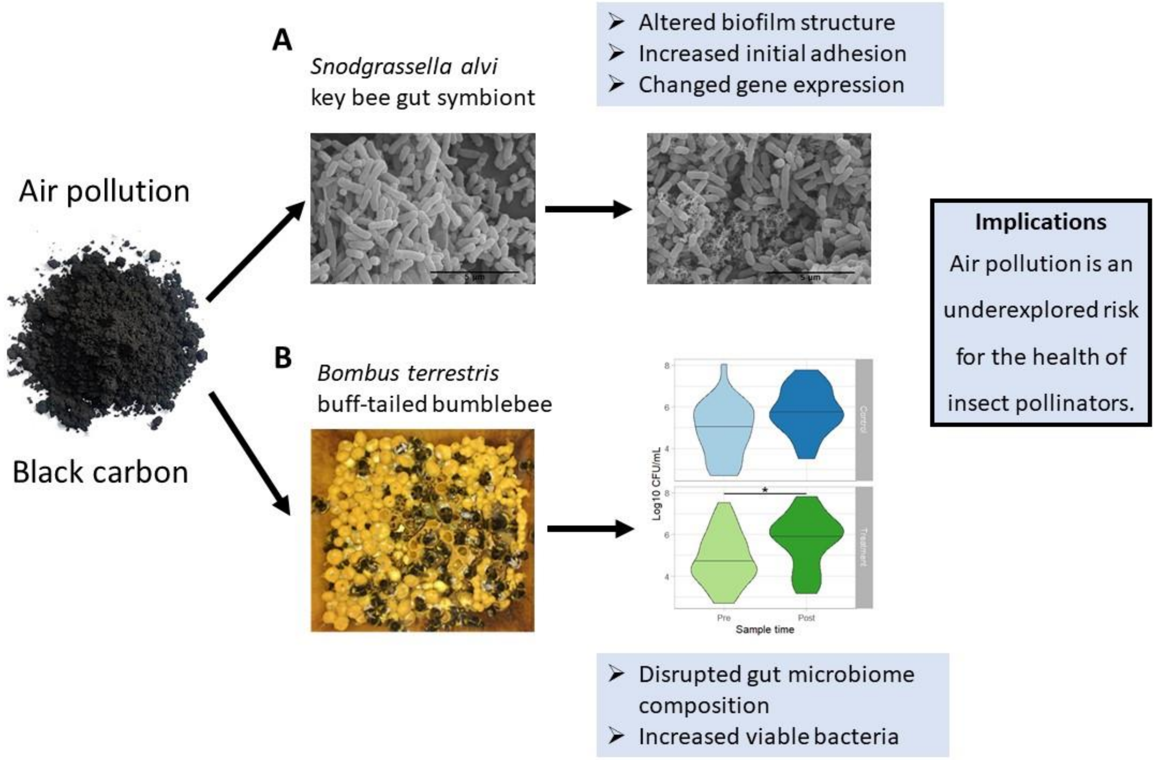

Supplementary Figure 1. Parallel experimental investigations to study the impact of particulate air pollution on the bumblebee gut microbiome. A) Major air pollution component black carbon alters adhesion, structure and gene expression of bee gut colonizer *Snodgrassella alvi in vitro.* B) Buff-tailed bumblebee (*Bombus terrestris*) exposed to black carbon, caused direct, measurable effects on the mature bee gut microbiome composition. The findings from these two in parallel investigations highlight the impact of air pollution directly on bacteria within beneficial microbial communities, and the potential health implications for essential insect pollinators.

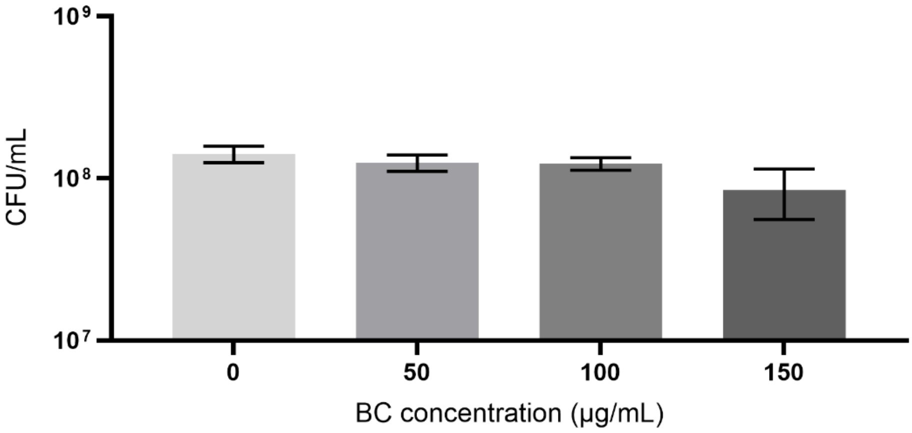

Supplementary Figure 2. Black carbon had no significant effect on *Snodgrassella alvi* growth after 24 hours of static incubation. Overnight cultures of *S. alvi* wkB2 were set to an OD of 0.02 in BHI and supplemented with 0, 50, 100 or 150 µg/mL black carbon (BC). Samples were incubated at 37 °C in 5% CO_2 conditions for 24 hours. Serial dilutions were plated onto blood agar and incubated for 48 hours to determine bacterial growth. A one-way ANOVA was performed finding no significant differences in CFU at the 0.05 level, n=3, error bars represent standard error of the mean.

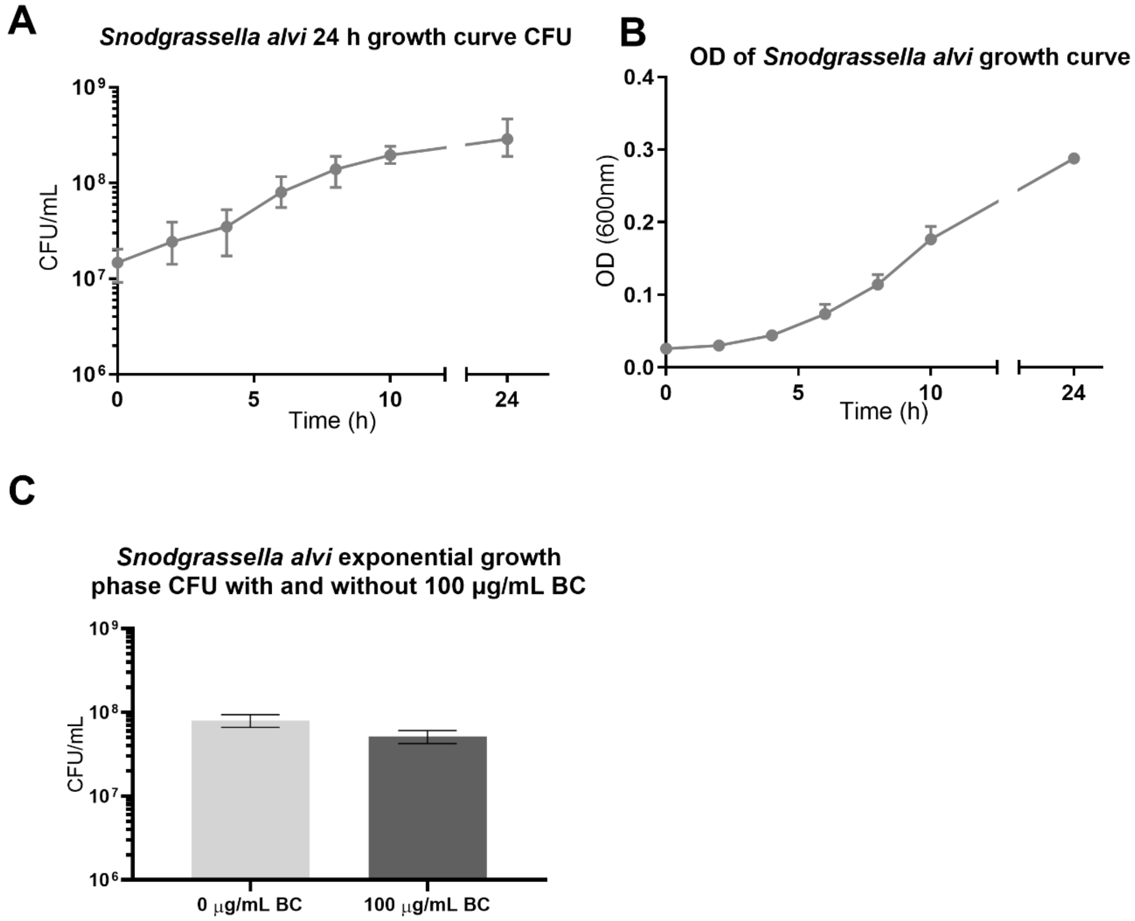

Supplementary Figure 3. *Snodgrassella alvi* growth curve and exponential growth CFU with and without black carbon. Overnight cultures of *S. alvi* wkB2 were diluted in fresh BHI to an OD of 0.02 and incubated at 37°C in 5% CO_2 conditions for 24 hours. At each timepoint samples were taken, serial diluted and plated in technical triplicate to determine CFU (A) and measured for optical density (B). Biological repeats n=4, means of CFU technical triplicates are plotted, error bars represent standard error of the mean. Exponential growth phase of *S. alvi* in these conditions was determined as an $OD_{600\ nm}$ of 0.08 (B). C) Black carbon (BC) had no significant effect on *S. alvi* exponential growth CFU ($t(6) = 1.70$, $p > 0.05$), biological repeats n=4.

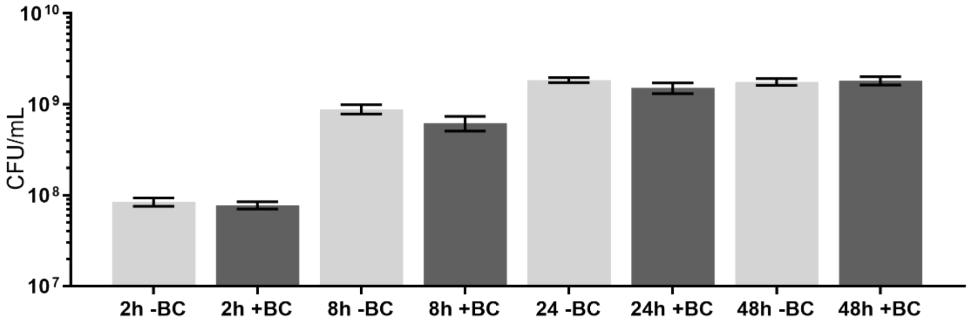

Supplementary Figure 4. The effect of black carbon on the total growth of *Snodgrassella alvi* biofilms. *S. alvi* wkB2 overnight cultures were diluted into fresh BHI with 0 (-BC) or 100 µg/mL black carbon (+BC), aliquoted into 12 well biofilm plates and incubated at 37° C in 5% CO_2 conditions for 2 hours, 8 hours, 24 hours or 48 hours. Biofilms were separated into fractions, serial diluted and plated to determine bacterial growth (fraction data presented in Figure 1A, 1B, 1C and 1D). Total CFU for each biofilm timepoint was combined, analysed with a one-way ANOVA and Tukey multiple comparison test finding no significant difference in total CFU with black carbon treatment for any timepoint at the 0.05 level.

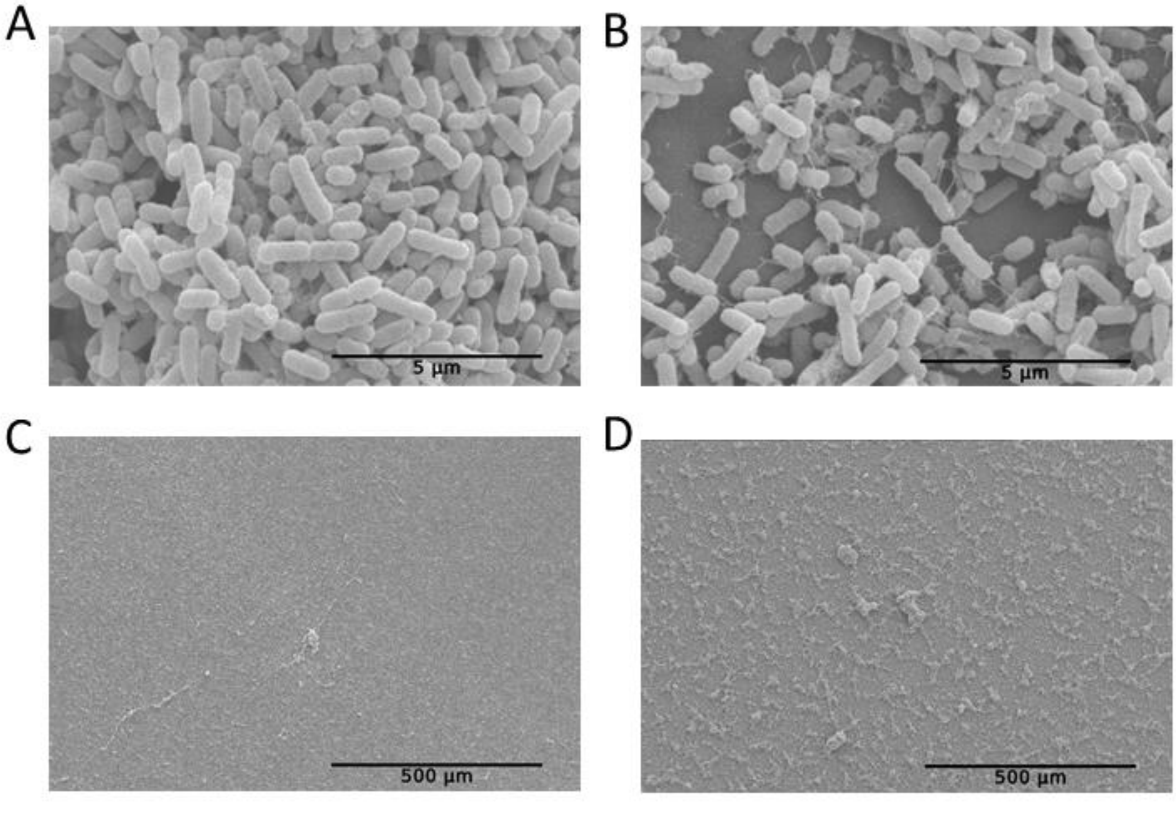

Supplementary Figure 5. The effect of particles on *Snodgrassella alvi* biofilm structure. Scanning electron microscopy images of 24-hour *S. alvi* wkB2 biofilms grown in BHI pre-treated media (A, C) or 100 µg/mL quartz (B, D).

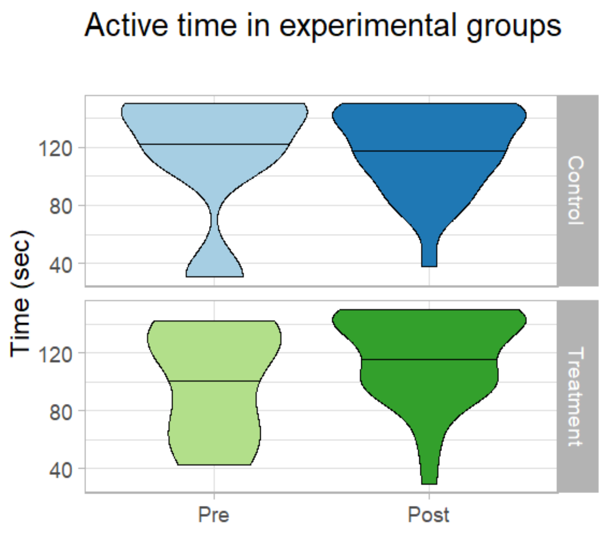

Supplementary Figure 6. The effect of black carbon on adult *Bombus terrestris audax* behaviour. Bees were randomly selected per experimental box (control n=6 boxes; black carbon treatment n=7 boxes), and active time was recorded for 150 seconds. This recording took place twice per day and was averaged to a daily mean time. Wilcoxon rank sum test with continuity correction was conducted on the daily mean active time within experimental groups Pre and Post treatment finding no significant difference in control or treatment experimental groups, lines represent median.

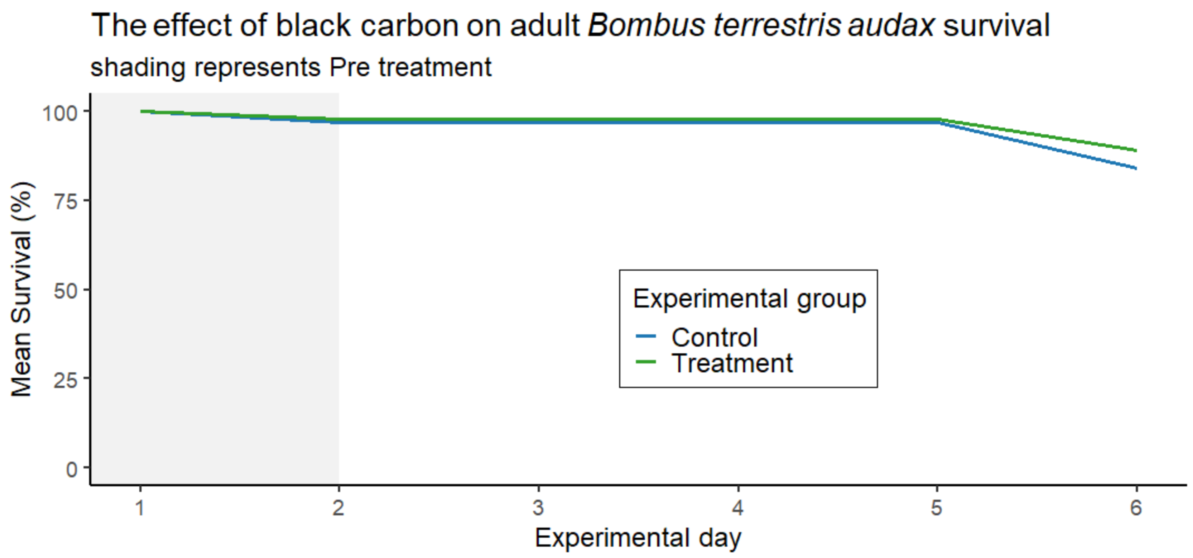

Supplementary Figure 7. The effect of black carbon on adult *Bombus terrestris audax* survival. Survival was recorded per experimental box on days two and six. Percentage survival was determined per box and the mean survival percentage was calculated from these values, finding no significant difference between control and black carbon treatment with the Wilcoxon rank sum test and continuity correction.

Supplementary Table 1. ANOVA comparison from a negative binomial regression of *B. terrestris* faecal CFU by experimental group, sample time and media.

Comparison	Chisq	Df	Pr(>Chisq)
Experimental group	6.31	1	**0.0120**
Sample time	10.90	1	**0.0009**
Media	3.07	1	0.0800
Experimental group:Sample time	20.22	1	**<0.0001**
Experimental group:Media	31.34	1	**<0.0001**
Sample time:Media	-351.83	1	1.0000
Experimental group:Sample time:Media	352.79	1	**<0.0001**

Supplementary Table 2. Post-hoc multivariate p value adjustment of Supplementary Table 1 ANOVA contrasts.

Treatment	Sample	Media	Contrast	Estimate	SE	Z ratio	p value
Control	Pre	.	MRS - Blood	-0.0839	0.449	-0.187	1.0000
Treatment	Pre	.	MRS - Blood	-0.9034	0.412	-2.193	0.2361
Control	Post	.	MRS - Blood	-0.1224	0.426	-0.287	1.0000
Treatment	Post	.	MRS - Blood	-0.3568	0.374	-0.953	0.9587
.	Pre	Blood	Treat - Cont	0.0637	0.393	0.162	1.0000
.	Pre	MRS	Treat - Cont	-0.7558	0.466	-1.622	0.6079
.	Post	Blood	Treat - Cont	0.3140	0.386	0.814	0.9826
.	Post	MRS	Treat - Cont	0.0795	0.415	0.191	1.0000
Control	.	Blood	Post - Pre	0.4475	0.411	1.089	0.9192
Control	.	MRS	Post - Pre	0.4090	0.463	0.884	0.9724
Treatment	.	Blood	Post - Pre	0.6978	0.367	1.903	0.4063
Treatment	.	MRS	Post - Pre	1.2443	0.419	2.971	**0.0315**

Where Sample=Sample time, Treat=Treatment, Cont=Control.
Significant differences at the 0.05 level are in bold.

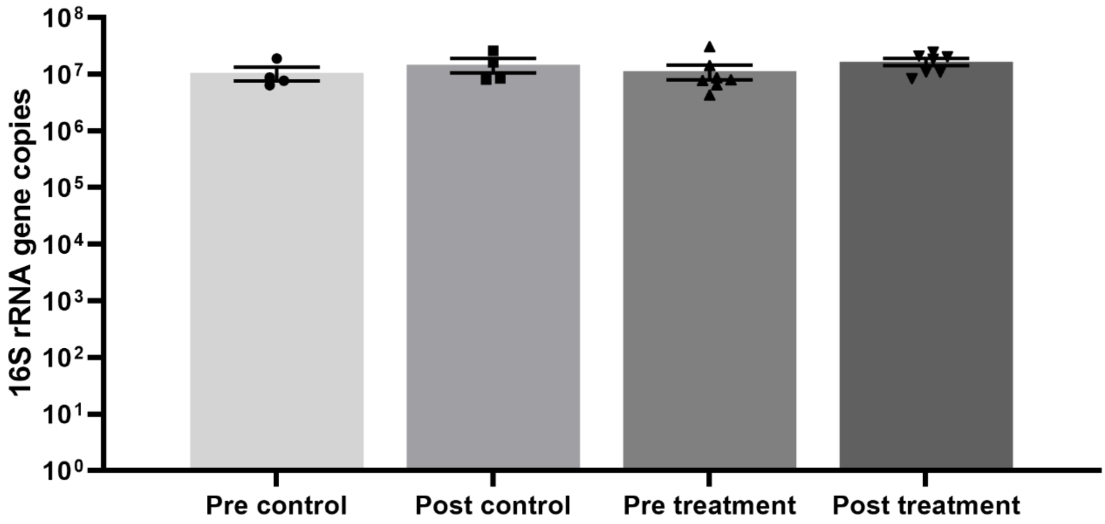

Supplementary Figure 8. No significant difference in total bacterial 16S rRNA gene copies between experimental groups. 16S rRNA copy number was amplified by qPCR in all samples using universal bacterial primers. Copy number per µL of sample was determined using standard curves from the amplification of the cloned target sequence in a pGEM-T vector of known concentration. A one-way ANOVA was performed finding no significant difference between experimental groups at the 0.05 level (control n=4, black carbon treatment n=7), error bars represent standard error of the mean.

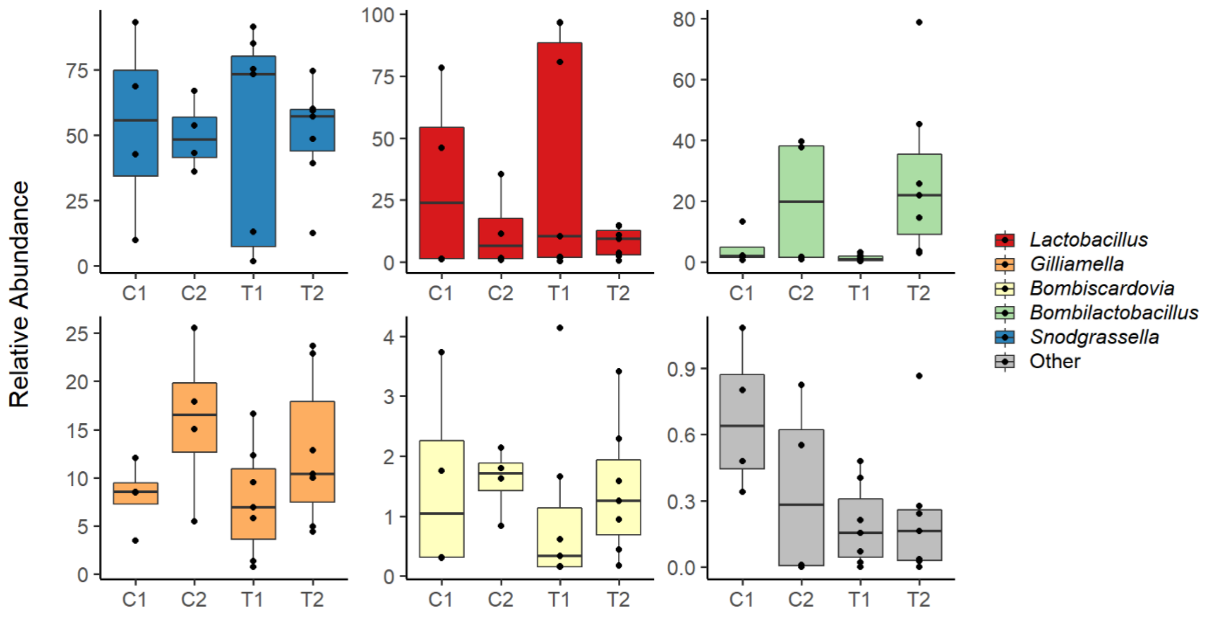

Supplementary Figure 9. The effect of black carbon on the relative abundance of the adult Bombus terrestris gut microbiome. Relative abundance of genera, displayed by experimental group (control n=4, black carbon treatment n=7). 16S rRNA amplicon sequences were assigned taxa using the BEExact database in Qiime2 and visualised with the phyloseq package in R. Where C1 = Pre Control, C2 = Post Control, T1 = Pre Treatment and T2 is Post Treatment.

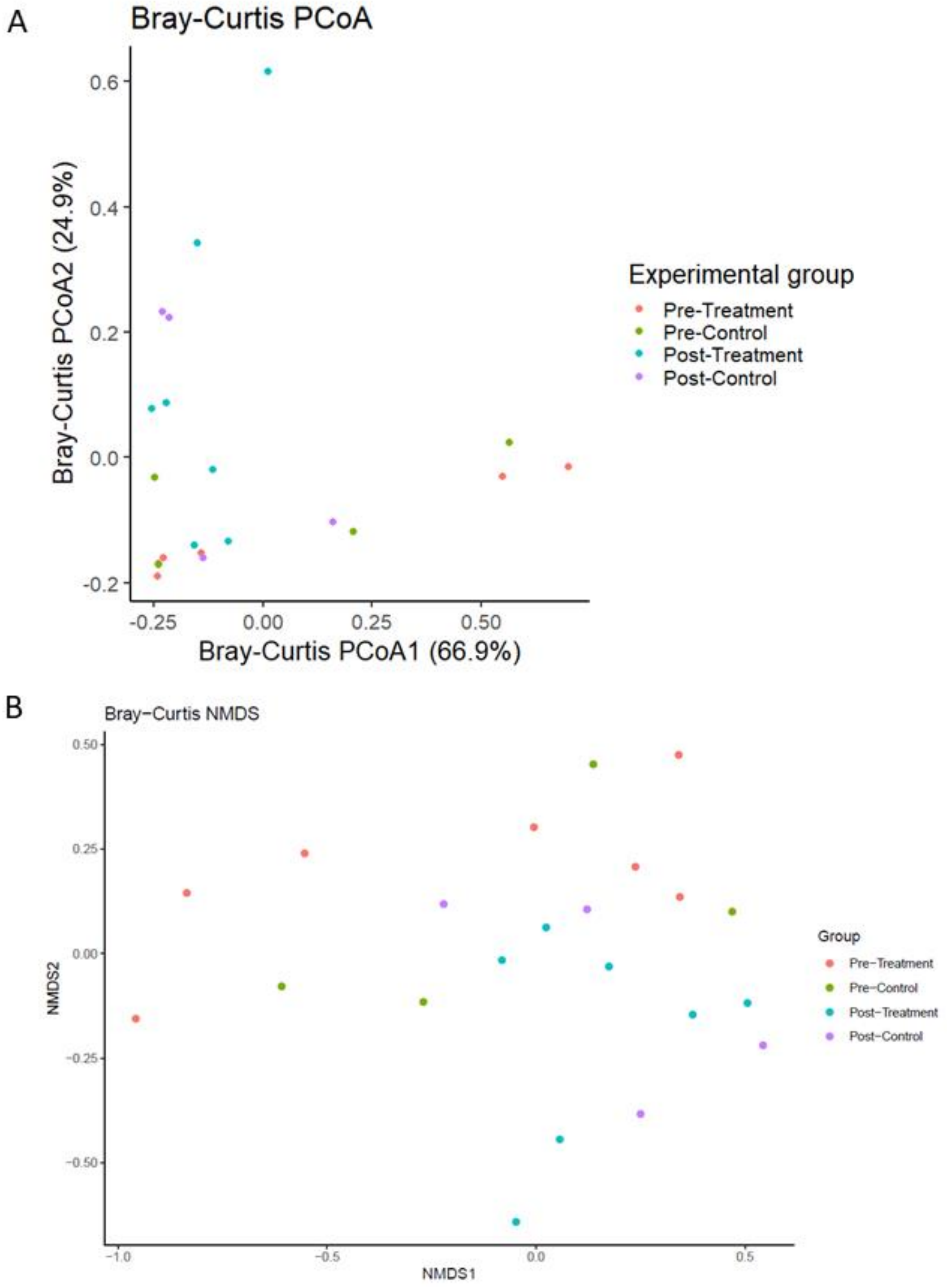

Supplementary Figure 10. The effect of black carbon on the beta diversity of the adult *Bombus terrestris* gut microbiome. Bray Curtis PCoA (A) and NMDS (B) showing microbiome dissimilarity by experimental group. PERMANOVA calculations found no significant differences between groups at the 0.05 level.

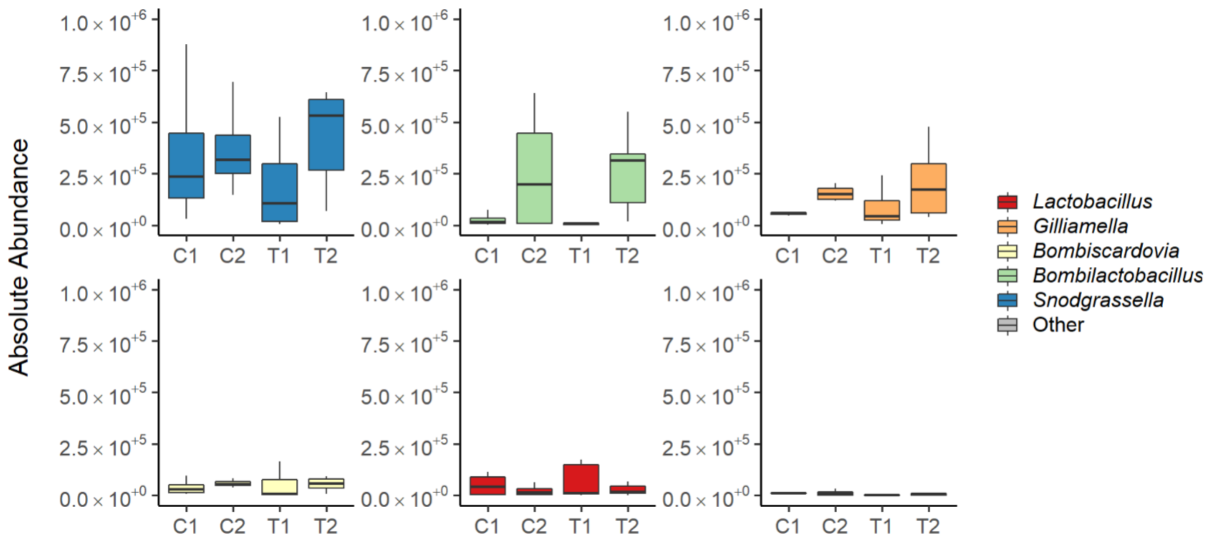

Supplementary Figure 11. The effect of black carbon on the absolute abundance of the adult Bombus terrestris gut microbiome. Absolute abundance of genera, displayed by experimental group (control n=4, black carbon treatment n=7). 16S rRNA amplicon sequences were assigned taxa using the BEExact database in Qiime2, adjusted for 16S rRNA copy number, and visualised with the phyloseq package in R. Where C1 = Pre Control, C2 = Post Control, T1 = Pre Treatment and T2 is Post Treatment.

Supplementary Table 3. Primers designed (*) and used in this study.

Gene name	Direction	Sequence 5' – 3'	Tm (°C)	Product length (bp)
LysR*	Forward	TACCCAGACCACTAGCCACC	60	140
	Reverse	CGAAGGCAACTGTATGCGTG		
PilD*	Forward	GAACATGAAGCACAGCGTCC	60	185
	Reverse	ATGATGGAACGAAGCTGGCA		
16S negative control*	Forward	GCAGCAGTGGGGAATTTTGG	60	138
	Reverse	GTACCGTCAGCACTAGGTGG		
16S rRNA copy number	Forward (28F)	GAGTTTGATCNTGGCTCAG	60	306
	Reverse (334R)	TGCTGCCTCCCGTAGGAGT		

$$\text{BC concentration (mg/g)} = \frac{\text{BC mass administered (0.105 mg)}}{\text{average weight of mouse (35 g)}}$$

$$\text{Feed concentration} = \frac{\text{BC concentration} * \text{average weight of bee}}{\text{Mass of daily intake of apiary solution per bee}}$$

$$\text{Feed concentration} = \frac{0.003\ \text{mg/g} * 225\ \text{mg}}{1.363\ \text{mg}}$$

$$\text{Feed concentration} = 0.495\ \text{mg/g}$$

Supplementary Figure 12. Calculation to determine the black carbon concentration to add to bumblebee feed. The dose of black carbon used was calculated using published exposures to humans (Chalvatzaki *et al.*, 2018) but accounted for bee dimensions, average daily consumption of apiary solution (Tyler *et al.*, 2006) and bee weight (Bumblebee.org, 2017). The concentrations used reflect those used in previous murine infection model and bee studies using apiary syrup and environmental metals that were adjusted to environmental exposure levels (Hladun *et al.*, 2016; Hussey *et al.*, 2017; Rothman *et al.*, 2019, 2019; Papa *et al.*, 2021; Seidenath *et al.*, 2023).

Supplementary Table 4. 16S rRNA copy number of core bee gut phylotypes.

Core bacterial species in dataset	16S rRNA copy number	*mean 16S rRNA copy number
Bombilactobacillus bombi	4	
Bombiscardova coagulans	3.5*	*Bifidobacterium* mean
Gilliamella bombi	4	
Gilliamella mensalis	4*	genus mean
Lactobacillus apis	4	
Lactobacillus bombicola	4*	*L. apis* and *L. panisapium*
Lactobacillus panisapium	4	
Snodgrassella alvi	4	
